# Impact of the Process Variables on the Yield of Mesenchymal Stromal Cells from Bone Marrow Aspirate Concentrate

**DOI:** 10.3390/bioengineering9020057

**Published:** 2022-01-29

**Authors:** Madhan Jeyaraman, Shiva Kumar Bingi, Sathish Muthu, Naveen Jeyaraman, Rathinavelpandian Perunchezhian Packkyarathinam, Rajni Ranjan, Shilpa Sharma, Saurabh Kumar Jha, Manish Khanna, Sree Naga Sowndary Rajendran, Ramya Lakshmi Rajendran, Prakash Gangadaran

**Affiliations:** 1 Department of Orthopaedics, Faculty of Medicine, Sri Lalithambigai Medical College and Hospital, Dr MGR Educational and Research Institute, Chennai 600095, India; madhanjeyaraman@gmail.com; 2 Department of Biotechnology, School of Engineering and Technology, Sharda University, Greater Noida 201310, India; saurabh.jha@sharda.ac.in; 3 Indian Stem Cell Study Group (ISCSG) Association, Lucknow 226010, India; skumarbingi65@gmail.com (S.K.B.); manishvenus@rediffmail.com (M.K.); 4 Fellow in Orthopaedic Rheumatology, Dr. RML National Law University, Lucknow 226010, India; 5 Department of Orthopaedics, Government Medical College and Hospital, Dindigul 624304, India; 6 Fellow in Joint Replacement, Department of Orthopaedics, Atlas Hospitals, Tiruchirappalli 620002, India; 7 Department of Orthopaedics, Government Medical College, Omandurar Government Estate, Chennai 600002, India; packkyarathinam@gmail.com; 8 Department of Orthopaedics, School of Medical Sciences and Research, Sharda University, Greater Noida 201310, India; orthoranjan04@gmail.com; 9 Department of Paediatric Surgery, All India Institute of Medical Sciences, New Delhi 110029, India; drshilpas@gmail.com; 10 Department of Orthopaedics, Prasad Institute of Medical Sciences, Lucknow 226401, India; 11 Department of Medicine, Sri Venkateshwaraa Medical College Hospital and Research Centre, Puducherry 605102, India; sowndaryasreeraj8@gmail.com; 12 Department of Nuclear Medicine, School of Medicine, Kyungpook National University, Daegu 41944, Korea; ramyag@knu.ac.kr; 13 BK21 FOUR KNU Convergence Educational Program of Biomedical Sciences for Creative Future Talents, Department of Biomedical Sciences, School of Medicine, Kyungpook National University, Daegu 41944, Korea

**Keywords:** bone marrow, bone marrow aspirate concentrate, mesenchymal stromal cells

## Abstract

Human bone marrow (BM) has been highlighted as a promising source of mesenchymal stromal cells (MSCs) containing various growth factors and cytokines that can be potentially utilized in regenerative procedures involving cartilage and bone. However, the proportion of MSCs in the nucleated cell population of BM is only around 0.001% to 0.01% thereby making the harvesting and processing technique crucial for obtaining optimal results upon its use in various regenerative processes. Although several studies in the literature have given encouraging results on the utility of BM aspiration concentrate (BMAC) in various regenerative procedures, there is a lack of consensus concerning the harvesting variables such as choice of anesthetic agent to be used, site of harvest, size of the syringe to be used, anticoagulant of choice, and processing variables such as centrifugation time, and speed. In this review article, we aim to discuss the variables in the harvesting and processing technique of BMAC and their impact on the yield of MSCs in the final concentrate obtained from them.

## 1. Introduction

Human bone marrow (BM) has been spotlighted as a promising source of mesenchymal stromal cells (MSCs) containing various growth factors and cytokines that are potentially utilized towards regenerative procedures involving cartilage and bone [1]. Following the harvesting and isolation process of the BM aspirate, the proportion of MSCs is only around 0.001% to 0.01% of the nucleated cell content of the bone marrow aspiration concentrate (BMAC) [2,3]. Hence, the quality and composition of the BM aspirate used in the preparation of the BMAC remain important to obtain optimal results upon its use in various regenerative procedures. Although the composition of the BMAC is largely dependent on the biological attributes of the patient concerned [4,5], the procedure variables such as location and technique of harvesting have a role in contributing to the variability in the yield of MSCs [6]. There is a wide range of devices and systems available to harvest and process BM aspirate, each using slightly different methods. However, the separation is usually based on the density gradient existing between the blood cells, platelets, nucleated cells, and serum proteins [7]. Based on the processing methods, the cellular and chemical composition of the BMAC may be altered, thereby leading to significant variation in their regenerative potential [8].

In BMAC, MSCs have the capacity to rebuild tissue by differentiating or inducing differentiation of native progenitors into a variety of cell types such as fibroblasts, chondroblasts, myocytes, and other forms of tissue-regenerating cells. BM also contains other multipotent cells, such as hematopoietic stem cells and vascular progenitors, which are likely to play a substantial role in the repair of damaged tissues. Various forms of clinical evidence exist to demonstrate the safety and efficacy of bone marrow-derived MSCs in an osteoarthritic (OA) knee [9,10,11,12,13]. The application of BMAC has been expanded to other indications such as a partial tear of ACL [14], meniscus injuries [15], tendon pathologies [16], bone defect in the form of delayed or non-union of fractures [17,18], chondral and osteochondral defects [19], and patellofemoral arthritis [3]. The components of BMAC are a significant amount of growth factors and cytokines in addition to MSCs and other multipotent cells. BMAC can be administered in isolation or as combination therapies in conjunction with platelet-rich plasma (PRP) [20], stromal vascular fraction (SVF) [21], or surgical procedures like core decompression [22], stress-relieving osteotomies [23], autologous chondrocyte implantation (ACI) [24], matrix-induced chondrocyte implantation (MACI) [24], and osteochondral autograft transfer system (OATS) [25]. Being a minimally invasive procedure, it is tolerated well by patients with better compliance with the post-procedural rehabilitation program. The short- and long-term results of BMAC in cartilage regeneration are encouraging with good to excellent clinical, functional, radiological outcomes [10,12,13]. No major adverse effects were reported with BMAC therapy except for donor site pain [26].

A fresh, uncultured, and unreduced volume of autologous BMAC injectate containing stromal cells are the potential regenerative and proliferative elements due to the synergistic coordination between the cellular elements and the pool of extracellular matrix, growth factors, and cytokines [27,28]. The modality of BMAC application can be given in the form of either intra-articular, intra-osseous, subchondral injections or surgical implantation with a bio-scaffold [29,30]. Among all the forms of injection, intra-articular modality remains relatively simple, easy to perform under sterile conditions whereas intra-osseous and subchondral injections require hospitalization as a daycare procedure. Due to the difficulty in accurate delivery of stromal cells into the lesion, engineered chondrogenesis came into existence which delivers a stable construct of stromal cells loaded along with bio-scaffold and growth factors [31]. Such a bio-scaffold reduces the chondrocyte loss, maintains the equal distribution of cellular structure, and enhances chondrogenesis [32]. The most commonly used bio-scaffold in the published literature are tricalcium phosphate, hyaluronate, collagen derivatives, agarose, fibrin glue, and chitosan [33,34]. Additive technology of autologous PRP or allogenic homologous platelet lysate has been admixed with engineered chondrogenesis technology to improve the clinical and functional outcome in OA knee individuals [35,36,37,38,39,40].

Several studies with the usage of BMAC for focal cartilage defects and OA knees have reported favorable outcomes. A single intra-articular BMAC injection was found to be a safe and reliable treatment option for grade 3 and 4 OA knees at 30 months follow-up [9]. Keeling et al. demonstrated improvement in pain and patient-reported outcomes in OA knee patients with BMAC injections at short- to mid-term follow-up. In severe degenerative arthritis, BMAC has shown clinical benefits compared to PRP and hyaluronate [12]. In a 5-year follow-up study, intra-articular BMAC injection has proven clinical benefits in K-L grade 1 and 2 osteoarthritis knees [41]. When admixed with an adipose tissue graft, BMAC has not shown superior results in OA knee individuals compared to BMAC alone [21]. In elderly individuals with OA knees, intra-articular BMAC has the potential to slow the timing for the arthroplasty procedure [42]. Gobbi et al.; demonstrated the complete healing of grade 4 multiple chondral injuries of the knee treated with autologous BMAC admixed with either collagen type 1 or 3. In second-look arthroscopy, hyaline-like cartilaginous tissues were demonstrated. These patients have shown no adverse events in a long-term follow-up [43]. Enea et al., have shown that scaffold-based BMAC along with microfracture to be a single-stage technique for the focal chondral defects of the knee [44]. With the MRI evidence, Krych et al. demonstrated that demineralized bone graft (DBG) admixed with BMAC improved cartilage filling in the focal cartilage defects when compared with DBG admixed with autologous PRP [45]. Conversely, a few studies have shown no significant benefits with BMAC when compared with either saline, placebo, hyaluronate, PRP, SVF, or clinical grade MSCs. No statistically significant difference was observed with autologous BMAC in terms of pain relief and functional improvement in patients with bilateral OA knee when compared with saline injections yet they claimed that BMAC was a viable cellular product for pain relief in a short term follow-up of 6 months [46].

Although several studies in the literature have analyzed the results of the utility of BMAC in various regenerative procedures with encouraging results [1,9,13], there is a lack of consensus concerning the harvesting variables such as choice of anesthetic agent to be used, site of harvest, size of the syringe to be used, anticoagulant of choice, and processing variables such as centrifugation time, and speed. These variables could be the source of the lack of consistency in the results of BMAC across the literature. Piuzzi et al.; performed a systematic review with 46 clinical trials, analyzing the preparation technique and usage of BMAC and demonstrated disparity in the preparation and utilization of BMAC. No definitive protocol could be derived out of these 46 clinical trials. On evaluating the protocols in 46 clinical trials, only 30% provided the quantitative metrics of the BMAC composition [6]. Murray et al. conducted a systematic review with 48 studies and found out the deficiencies in the BMAC preparation protocol and BMAC composition [47]. None of the 48 studies included provided a proper technique or protocol to standardize BMAC formulations [47]. Hence, there is a lacuna in the literature on the standardized method of harvesting and processing of the BMAC to obtain optimal results [6].

## 2. Variables in the Harvesting and Processing Technique of Bone Marrow Aspiration Concentrate (BMAC)

**Figure 1:**
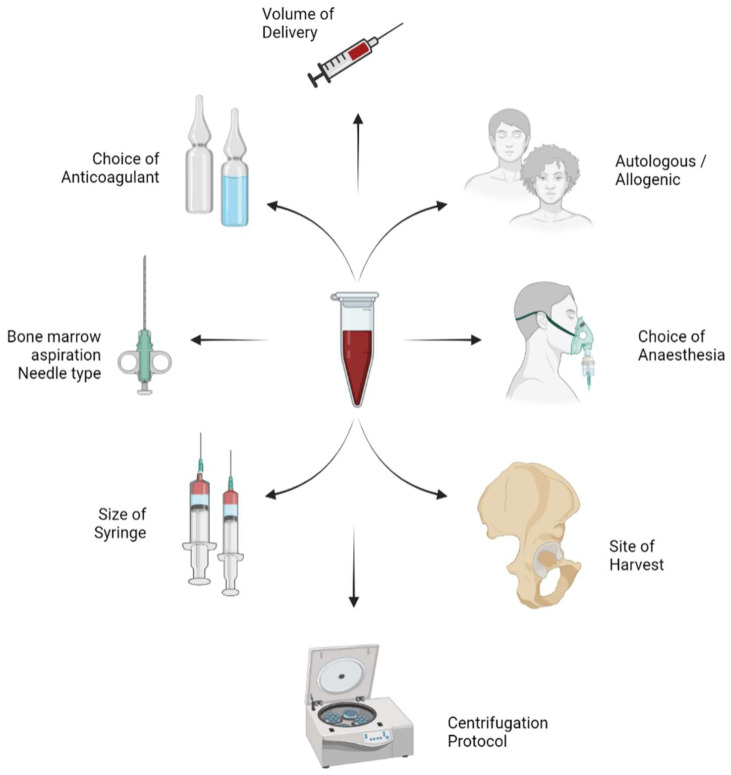
Variables in the harvesting and process technique of bone marrow aspiration concentrate (BMAC).

In this review article, we aim to discuss the variables in the harvesting and processing technique of BMAC as shown in Figure 1 and their impact on the yield of MSCs in the final concentrate obtained.

### 2.1. Autologous versus Allogenic Mesenchymal Stromal Cells (MSCs)

The efficacy of BMAC in cartilage regeneration depends upon the ability of the cells in the BMAC to withstand and restore the biochemical disharmony in the pathological milieu being evaluated [48,49]. Autologous sources of MSCs from bone marrow are relatively easy to harvest, cost-effective without the risk of any graft rejection or disease transmission [50]. However, autologous MSC products need a two-staged procedure for cartilage regeneration while planning for culture expansion whereas allogeneic MSC preparations can be delivered as a single staged procedure with the desired dosage of MSCs [51]. Allogeneic products namely CARTISTEM (allogeneic cord blood-derived MSCs—2.5 × 10^6^ cells/500 μL/cm^2^ area of knee cartilage) [52], Stempeucell (allogeneic ex-vivo cultured pooled human BM-MSCs—2 × 10^8^ cells cryopreserved and stored in 15 mL cryo-bags) [53], and JointStem (autologous AD-MSCs—10 × 10^7^ cells) [54] launched the MSC-derived products with a definite dosage for cartilage injuries. Nonetheless, allogeneic MSC preparation lacks literature evidence in terms of long-term safety, and efficacy [55]. The dynamic fate of implanted allogeneic MSCs is under debate.

Due to cellular heterogeneity and the subjective characteristics of the MSCs harvested, autologous sources of the MSC cocktail generate inconsistent results upon the analysis in varied scenarios whereas the allogeneic pool of MSC contains homogeneous cells with theoretical grounds to deliver consistent results although they also suffer from immune reactions [55,56,57]. Hence, the clinical outcome depends on the harvesting and cultural characteristics of MSCs isolated either from the autologous or allogeneic source. As age progresses, the MSC count in autologous sources decreases [58]. To achieve the desired cartilage regeneration by MSCs, allogeneic sources of MSCs can be isolated and culture-expanded to provide more MSCs and to provide off-the-shelf products to allow for emergency application. To have the desired cartilage regeneration, the optimal dose of cells, adjuvants, and source of MSC harvest has not been standardized.

Cell-based regenerative therapies rely on consistent, potent, and effective sources of MSCs. Various researchers have conducted clinical trials on embryonic stem cells and induced pluripotent stem cells (iPSCs). The parameters affecting the commercialization of iPSCs are cost, long-term culture and storage, and tumorigenic potential [59]. Autologous cell sources avoid host immune rejection for cell engraftment and retention. Autologous cell therapy requires multi-stage procedures for cell isolation, expansion, and transplanting back to target sites. The variability in the clinical outcome by using the autologous cell source depends on the subjective patient difference which is a major obstacle for reliability and quality control of the product [50]. To overcome all these obstacles, an allogeneic source of cells can be attempted as promising next-generation cell therapy.

Before selecting any allogeneic MSC product for cartilage regeneration, certain parameters should be considered such as MSC passage number, the desired number of MSCs to be injected, the immunogenicity of allogeneic MSCs, the shelf life of allogeneic MSC product, and injection and rehabilitation protocols [57]. Allogeneic preparation of MSCs has to be carried out in a Good Manufacturing Practice (GMP)-certified laboratory with the due regulatory guidelines and protocols [57]. The double negative cell population (HLA-1 negative and HLA-2 negative) renders an allogeneic pool of MSCs as the preferential therapeutic product in the field of regenerative medicine [60]. The temporal relationship between the efficacy of the MSCs delivered in cartilage regeneration with the number of cellular passages, dosage and frequency, cell of origin, usage of scaffolds, and bio-micromolecules has yet to be established. Having discussed the yield, safety, and efficacy of both the autologous and allogenic sources of MSCs, the final choice is always taken upon discussion with the patient based on the affordability and personal preferences.

### 2.2. Choice of Anaesthesia

The available literature lacks the standard guidelines or consensus for reducing the pain during bone marrow aspiration (BMA) and the post-procedural period. The pain experienced by more than 50% of the individuals who are undergoing BMA was neglected [61]. There are both dependent and independent factors in experiencing pain during bone marrow aspiration namely age, gender, body mass index (BMI), information regarding the procedure, previous BMA, site of BMA, the experience of the physician or surgeon, duration of BMA, and the level of difficulty in performing BMA [62]. A temporal association has been documented between duration and the level of difficulty in performing BMA [63]. The patients undergoing repeated BMA procedures were reported to endure unbearable pain [64]. An adequate level of anesthesia and analgesia is essential for any orthopedic surgery to be successful. For successful harvesting of bone marrow, monitored anesthesia (conscious sedation), regional anesthesia, and general anesthesia are the most widely used anesthesia techniques [62,65,66].

Various studies have used different local anesthetic agents [lidocaine, chloroprocaine, bupivacaine, articaine, or mepivacaine) to aspirate BM but reported no significant difference in the reduction of pain [67,68]. The coupling of intravenous sedation (IVS, lorazepam, midazolam, or diazepam) with local anesthesia reduces anxiety and pain perception [69,70] and IVS drugs also cause retrograde amnesia in some patients [71]. Holdsworth et al. documented less BMA pain and distress in patients receiving propofol/fentanyl general anesthesia than the eutectic mixture of local anesthetics (EMLA) or oral midazolam/EMLA [72]. Pretreatment with oral tramadol 50 mg 1 hour before BMA reduces procedural pain significantly while compared with oral placebo [63]. General anesthesia by propofol and fentanyl offers a good choice for short-term painful procedures in children undergoing treatment for BMA as a daycare procedure [66]. Deep sedation with midazolam, fentanyl, and propofol has proven benefits in the form of pain reduction [73]. The literature lacks the association between the choice of anesthesia and the yield of BMAC. Further studies are warranted to validate the yield of BMAC with the different choices of anesthesia used for the BMA procedure.

### 2.3. Site of Aspiration

Bone marrow and adipose tissue remain the commonly investigated source of MSCs for cartilage regeneration. The number of MSCs present in bone marrow is less when compared with adipose tissue [74]. The anterior and posterior iliac crests [8,75,76], the ilium [77,78], the proximal humerus [78,79], the proximal tibia [65,80], the distal femur [81,82], the distal tibia [83], the sternum [84,85], the mandible [86,87], and the calcaneum [88,89] are the most common locations for BMAC harvest without significant morbidity to the donor site. The primary site recommended to harvest BMAC is the iliac crest. Hernigou et al., defined the “zone model” and “sector rule” in pelvic bone for choosing the entry point to draw bone marrow [90]. This zone model divides the iliac crest into six different zones as sectors. With the help of CT scans, the sectors were defined based on the bone thickness, the maximum available bone depth for trocar purchase in these different parts of the iliac crest, and the corresponding vascular structures at risk. Hernigou et al., determined that the safe entry point should be approximately 2.5 cm distally from the anterior superior iliac spine (ASIS) [90]. Various studies have stated that BMAC is harvested from ASIS or the iliac crest in the supine position and posterior superior iliac crest (PSIS) in the prone position, respectively [8]. In children, the sternum, anterior iliac crest, tibia, and spinous process of the vertebra are the most common sites for bone marrow aspiration [76]. Bierman et al., reported that the posterior iliac crest is the safest site for harvesting bone marrow as it is the thickest portion of the posterior segment of the iliac crest with a huge amount of cancellous bony tissues [76]. The volume of bone marrow in the posterior iliac crest is more than the anterior iliac crest [76].

There is a longstanding ongoing debate on the harvesting technique of BMAC. Oliver et al. [91] stated that more BMAC cellular concentrations can be obtained by single insertion technique than multiple insertion technique whereas Peters et al. [92] commented that multiple insertions (up to four) resulted in a higher volume and concentration of BMAC cellular components. Kasashima et al. stated that the insertion of 5 mm of bone marrow needle into the equine sternum three times yielded more BM-MSCs with reduced peripheral blood contamination [93]. They further claimed that under USG guidance, the accurate placement of bone marrow needles into the medullary cavity facilitates the harvest of bone marrow with the least possible damage to the sternum [93].

### 2.4. Syringe Used

The quantification of mononuclear cells provides an approximate estimate of the actual number of mesenchymal stromal cells in bone marrow aspirate. The proportion of MSCs lies between 1 per 10^4^ to 1 per 10^6^ mononuclear cells in BMAC [94]. The age of the individual remains a major factor affecting the proportion of MSCs in the BMAC [95,96]. Since dilution of the BMAC with blood is also a significant factor that reduces the MSC content, the technique of aspiration plays a role in the MSC yield [97,98]. There is an artificial increase in the number of mononuclear cells (MNCs) with the dilution of aspirate with blood without any increase in the MSCs due to their paucity in the blood [97,99]. The technique to avoid such dilution is to keep the depth of the needle in the central region of the iliac marrow that lies between the subendosteal and perisinusoidal regions. Further advancement into the sinusoidal regional results in significant dilution of the aspirate with blood. Hence, small aspirates of 1 to 4 mL obtained with a 10 mL syringe have been proposed and described as a standard technique to avoid blood dilution although it increases the time required to obtain sufficient volume of the aspirate [5,100].

Some surgeons prefer a larger-volume syringe to improve the rate of bone marrow aspiration. The rationale behind the use of a larger-volume syringe is that it can generate a stronger negative pressure and, therefore, aspirate more MSCs with a larger overall aspirate volume. According to the findings of the study conducted by Hernigou et al. [97], bilateral aspirates were collected from the iliac crest of the same patients using a 10 mL syringe and a 50 mL syringe, respectively. The frequencies of MSCs as defined by the total cell count, the progenitor cell concentration, and the number of colony-forming units obtained from each size of the syringe were analyzed. All of the bone marrow aspirates were obtained from the same surgical procedure. The findings revealed that bone marrow aspirates taken using a 10 mL syringe contained higher concentrations of MSCs than comparable controls taken with a 50 mL syringe. The concentrations of progenitor cells were on average 300% greater when using a 10 mL syringe compared to matched controls when using a 50 mL syringe [97].

According to the equation, pressure = force/area, a smaller diameter generates higher pressures with the same force. Hence, a smaller diameter syringe can generate a stronger negative pressure with the same force. For an equivalent force of a draw, the negative pressure exerted by the syringe is stronger with a small diameter plunger than with a big diameter plunger. Also, it is easier to draw the plunger of a small syringe at a higher pace as compared with a large syringe due to lower drag. The ease of drawing a small syringe provides higher transmission of force to the plunger during the aspiration. Friction also will slow down the pace that the plunger can be pulled. It is likely that there will be two major components to this friction: friction created by the plunger seal as it passes through the barrel and drag caused by the fluid itself. When dealing with fluid as viscous as bone marrow, the second component might be rather substantial. A plunger with a smaller diameter reduces frictional resistance as it moves because it provides a smaller area for the frictional forces to impose resistance on the plunger. Hence, 10 mL syringes seem more ideal for a better yield of MSCs from the BM aspirate [97].

### 2.5. Bone Marrow Aspiration Needle Type

The yield of bone marrow to prepare BMAC is very important in order to deliver the MSCs with appropriate quality and quantity. The bone marrow aspiration needle plays a major role in harvesting the bone marrow to deliver a higher number of progenitor cells to observe a better clinical and functional outcome. There are various bone marrow needles available on the market namely (a) Jamshidi needle, (b) Modified Jamshidi needle, (c) Klima sternal needle, (d) Salah bone marrow aspiration needle, (e) Watherfield iliac crest bone marrow aspiration needle, and so on [84]. Feddahi et al., observed no significant difference regarding the quantity of MNCs and regenerative potency of MNCs in BMAC for either the Jamshidi needle (JAM) on one side and the Marrow Cellution^®^ Needle (AMC) on the other side when aspirated from the posterior iliac crest of 12 patients [101]. Oliver et al. reported that a slightly higher quantity of MNCs was harvested by multiple-site (31 × 10^6^ mL) than single-site (23 × 10^6^ mL) bone marrow aspiration using the standard Jamshidi needle whereas a higher quantity of MSCs was retrieved using the single-site method (3486/mL concentrate) than the multiple-site method (2722/mL concentrate) [91]. Recently there have been many advocacies for aspiration needles with the lateral aspiration ports to enhance a circumferential retrieval of BM and with a claim to reduce the cellular lysis due to the procedure. We did not find any literature supporting their superiority over the conventional aspiration needles to date.

### 2.6. Anticoagulant of Choice

Although the impact of anticoagulants that are used during the harvesting of BM is negligible we still wanted to consider them as a variable since we noted heterogeneity among the studies available in the literature. There are many anticoagulants in the clinical practice for harvesting blood and bone marrow, namely acid citrate dextrose (ACD), citrate-phosphate-dextrose (CPD), ethylenediaminetetraacetic acid (EDTA), heparin, and so on. A few studies have shown that mere rinsing of the bone marrow needle and syringe with heparin before bone marrow harvesting was sufficient [102], whereas a few studies stated the usage of 100 IU of heparin per ml of bone marrow to achieve sufficient anticoagulation [103]. Moreover, the published literature has demonstrated convincing evidence on heparin, although it belongs to bio-active substances which can potentiate the biological effects of protein binding and the sustained release of growth factors and cytokines [104,105].

Preclinical evidence of co-administration of BM-MSCs and heparin resulted in the prevention of clotting of BM-MSCs. All the studies have proven that heparin has facilitated the proliferation of MSCs both in vitro and in vivo [106,107,108,109]. An in vitro study by Simann et al. [110] with the usage of 20 U/mL unfractionated heparin facilitates osteogenic response when used along with BM-MSCs whereas Ling et al. [111] reported a detrimental effect on trilineage differentiation when BM-MSCs are co-cultured along with 160 ng/mL of heparin. Kim et al. [106] commented that heparin should not be co-cultured with MSCs as a culture supplement. Various sources of MSC origin such as bone marrow, adipose tissue, and umbilical cord were isolated and cultured along with human platelet lysate with or without heparin. Transcriptome analysis revealed the gene regulation depending on the origin of stromal cells. They suggested that heparin did not affect the long-term proliferation and trilineage differentiation of MSCs [112]. No similar analysis was performed on other conventionally used anticoagulants such as ACD, CPD, EDTA to give any comparative benefit of one over the other.

### 2.7. Ideal Centrifugation Speed and Time

United States Food and Drug Administration (US-FDA) has approved the usage of autologous, uncultured, and unreduced BMAC as a means of MSC therapy for various indications. After aspirating from the source, bone marrow has been subjected to density gradient centrifugation to separate progenitor cells from the cellular mixture of red blood cells, granulocytes, immature myeloid precursors, and thrombocytes. In BMAC, there is a considerable concentration of growth factors, cytokines, and bone morphogenic proteins (BMPs) that are anti-inflammatory and anabolic in nature. BMAC has been reported to have a higher concentration of interleukin-1 receptor antagonist (IL-1Ra) which counterbalances the catabolic effect in inflammatory disorders and provides better pain relief. Various studies have obtained variable amounts of BMA ranging from 30 mL to 120 mL from different sources [42,113,114]. Chahla et al., obtained 60 to 90 mL of BMA and processed with dual centrifugation of 700 g for 10 min and 1400 g for 6 min to procure 6 to 8 mL of BMAC [8]. Themistocleous et al., obtained 20 mL of BMAC from 80 mL of BMA by single spin centrifugation at a rate of 960 g for 15 min [9]. Single spin low centrifugational force of 580 g maximizes stromal cell viability and integrity and yielded optimal separation between bone marrow layers [9].

To improve the separation techniques of BMAC from BMA, various researchers have used fixed density solutions such as Ficoll Hypaque or Lymphoprep™ with a density of 1.077 g/mL to separate the mononuclear cells from the red cell layer at different centrifugation forces (range: 2200 g to 3200 g) and different dilution ratios (1:1 to 1:3) [115,116]. However, the optimal concentrate of BMAC could not be standardized due to the presence of numerous variables such as cellularity, nature of bone marrow (yellow or red), and viscosity. Due to the different centrifugal forces, the rate at which the cells’ sediment and cellular viability and integrity may differ and provide a difference in the clinical results. Naung et al., concluded that more number functional osteoprogenitor cells (mean ± SD: 6.87 × 10^7^ ± 4.84 × 10^7^) were yielded with lower centrifugation force (400 g) with an equal dilution of 1:1 than higher centrifugation force (1000 g) with 1:3 ratio dilution [117]. Estrada et al., prepared BMAC through density gradient centrifugation by Ficoll-Paque Premium at a rate of 800× *g* for 25 min [118]. If conventional methods of preparation of BMAC are employed, the standard prescribed centrifugation protocol preferred by the manufacturer of the fixed density reagent would be sufficient to separate the MNC layer from the red cell layer, thereby ensuring a better yield of MSCs.

### 2.8. Volume of Delivery

The literature lacks the documentation of a standardized volume of BMAC to be injected, quantification of the MSCs, and the ideal number of BMAC injections needed for cartilage regeneration since these depend on subjective factors such as stage of the disease, the yield of the MSCs from the patient, and proliferation potential of the MSCs in the BMAC. Most recent studies have reported aspirating 30 to 120 mL of BMA to prepare 10 to 12 mL of BMAC for clinical applications [3,9,30,42,119,120,121,122,123,124,125]. Various studies have shown excellent clinical and functional outcomes with single dose or multiple doses of BMAC ranging from 4 to 12 mL per knee for cartilage regeneration [30,119,122,123,124,126]. A single dose of 8 mL BMAC demonstrated a superior clinical outcome than PRP and autologous conditioned serum in the patients with OA knee [12]. However, it has now been established that it is not the volume of delivery that matters but it is the count of active cellular components in the vehicle used for delivery that brings about a significant change in the perceived outcome [119]. Recently, the MNCs have been suspended in growth factor-rich serum-based delivery vehicles such as PRP to have an additive effect on the regenerative potential of the BMAC.

## 3. Authors Perspective

**Figure 2:**
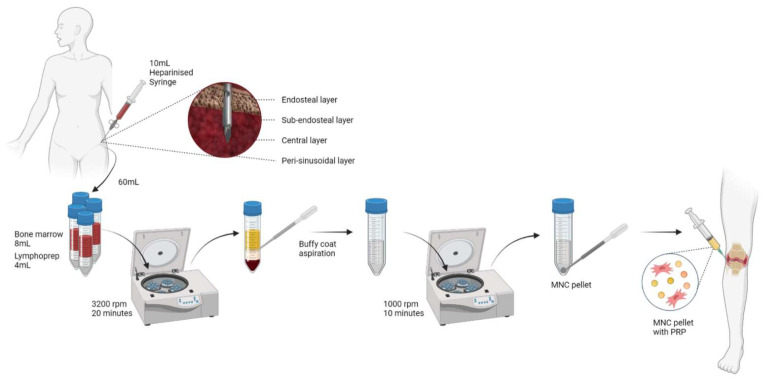
Bone marrow aspirate concentrate preparation process.

Considering the available variables in the harvesting and processing of the BMAC, the authors perform the procedure as a daycare procedure using an autologous source of MSCs following all the sterile aseptic precautions under LA and IVS. The conventional harvesting site preferred by the authors remains 2.5 cm distal to the ASIS as described by Hernigou et al. We used 10 mL syringes prefilled with 1000 U of heparin/10 mL for aspiration of BM from the central region of the iliac marrow that lies between the subendosteal and perisinusoidal regions using an 11-gauge bone marrow needle with terminal and lateral fenestrations, as shown in Figure 2 [8]. We employ multiple (2–3) sites for aspiration of BM. We use the density gradient separation technique with the fixed density solution of 1.077 g/mL to separate the red cell layer and the mononuclear layer at centrifugation force recommended by the manufacturer at 3200 rpm for 20 min [127,128]. The supernatant MNC layer is subjected to a second centrifugation at 1000 rpm for 10 min to obtain the MNC sediment. We re-suspend the mononuclear cells in 5 mL of PRP and administered them by intra-articular route to the target joint [20]. Apart from the assessment of the outcome of the procedure based on the patient-reported outcomes such as pain relief and functional improvement, the authors recommend the critical assessment of outcome of the procedure by peers to avoid reporting and observer bias.

## 4. Conclusions

BMAC contains MSCs and several growth factors and cytokines with established paracrine and immunomodulatory effects thereby making them a promising biological tool in the management of knee osteoarthritis. Studies reported to date have given encouraging results on the clinical improvement rather than the quality of the regenerate attained due to the intervention. Moreover, significant heterogeneity exists among the studies that preclude a direct comparison of their results. Although several variables exist on the harvesting and processing of BMAC, standardization of the BMA procedure with sound literature background is required. Available evidence on the identified variables has been comprehensively presented here. Future studies focusing on analyzing the role of the individual variables identified in the review would throw some light on the standardization of the procedure in order to attain comparable results across the studies conducted using them.
